# Allocation of Pediatric Home Care Nursing Hours: The Minnesota Experience

**DOI:** 10.1097/NHH.0000000000001035

**Published:** 2022-01-07

**Authors:** Lindsey Paitich, Chris Luedemann, Judy Giel, Roy Maynard

**Affiliations:** **Lindsey Paitich, BSN, RN**, is Director of Home Care Nursing, Pediatric Home Service, Roseville, Minnesota.; **Chris Luedemann, MD, BSN, RN**, is Former home care nurse, Pediatric Home Service, Roseville, and Resident, Radiology, University of Minnesota, Minneapolis, Minnesota.; **Judy Giel, RRT**, is Chief Clinical Officer, Pediatric Home Service, Roseville, Minnesota.; **Roy Maynard, MD, FAAP**, is Medical Director, Pediatric Home Service, Roseville, Minnesota.

## Abstract

Despite an increasing demand for pediatric home care nursing, there is no comprehensive or universal standard of care for prescribing pediatric home care nursing hours based on a child's medical complexity. Adoption of a qualification tool (QT) to allocate home care nursing hours based on the medical complexity of a child may mitigate inequality in access to care and improve the patient and family experience. A QT, developed in Minnesota, recommends home care nursing hours based on the level of medical complexity and need for skilled nursing interventions. Four hypothetical case studies demonstrate the use of the QT to calculate recommended nursing hours. To validate the tool, a survey of discharge planners found a percentage difference in calculated hours of 4.1, 5.7, 11.2, and 24.9 in the four case studies. Discharge planners rated the usability of the QT as favorable with a score of 3.6 on a Likert scale of 5. The recommended nursing hours prescribed for families, based on the QT, was perceived as meeting the needs of the child by 56% and 42% of surveyed parents and home care nurses (HCNs), respectively. The need for additional nursing hours was expressed by 33% and 50% of parents and nurses, respectively. In general, HCNs' assessment of allocated nursing hours paralleled that of parents. Further refinement and adoption of a standardized QT to allocate home care nursing hours may improve access and outcomes for children requiring home care nursing.

**Figure:**
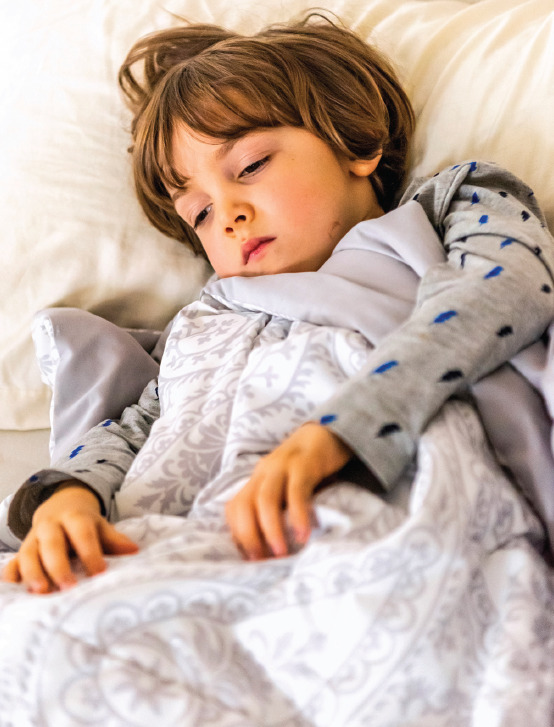
No caption available.

The population of children with medical complexity (CMC) has increased in Minnesota (Muesing et al., 2020) as well as nationally (Burns et al., 2010). This trend has resulted in demands to shift pediatric complex care from hospital to home. Consequently, the most fragile and technology-dependent of CMC will receive pediatric home care nursing, with the greatest need for children <5 years of age (Benneyworth et al., 2011). Home care nursing facilitates a safe and timely transition to home, minimizes family burnout, and may decrease costly hospital readmissions (Gay et al., 2016). However, a shortage of pediatric home care nurses (HCNs) (Sobotka et al., 2019) may delay hospital discharge (Maynard et al., 2019).

Home care services are received by 5% to 7% of pediatric hospital discharges (Berry et al., 2016; Feudnter et al., 2005). There are significant state variations in use of posthospital pediatric home care nursing, which may contribute to healthcare disparity (Rasooly et al., 2020). Currently no standard exists for prescribing pediatric nursing hours based on the medical complexity of the patient (Sobotka et al., 2020). Due to the limited supply of HCNs, there is a need for an objective method for allocating home care nursing hours based on skilled nursing interventions and patient complexity. Assignment of HCNs to low-acuity patients limits their availability for more complex patients.

In Minnesota, some home care agencies have been using a qualification tool (QT) to recommend nursing hours to a patient's physician. If approved by the physician, a request for prior authorization is submitted to insurance. Ultimately, insurance decides whether to approve or deny nursing hours based on the patient's plan and benefits. This article describes the history, development, and application of a QT to calculate nursing hours using four hypothetical case studies modified from real-life patients.

## Historical Perspective

In 1982, the Katie Beckett waiver was passed allowing Medicaid to cover services for children in the home independent of parent's income if the care was less expensive than hospital care (Koop, 1986). As a result, pediatric home care became more prevalent, payments increased, and some initiatives were undertaken to improve the quality of home care for children. A concern was raised in 2008 by Minnesota community physicians caring for homebound technology-dependent children, identifying an unexpected increase in unplanned readmissions, morbidity, and mortality.

In 2009, 6 out of 15 invited Minnesota nursing agencies agreed to participate in monthly collaborative meetings to focus on the development of consistent training requirements and standards of care for pediatric HCNs. A Minnesota Department of Human Services survey at that time identified that 43% (336/781) of pediatric patients received services from the six participating nursing agencies. Minnesota Medical Assistance (MMA), Minnesota's Medicaid program, as well as state waivers funded 70% of these patients. To receive state reimbursement, nursing agencies filed an MMA Home Care Nursing Assessment Form. Discrepancies associated with this document included a lack of objectivity, no tool for allocation of nursing hours based on medical complexity, and failure to address the home environment or family capacity to learn and perform complex health-related tasks. There was a need to better determine the intensity of nursing care needs and standardize recommendations for nursing hours from different perspectives.

Collaboration was expanded to include community stakeholders including home medical equipment suppliers, physicians, hospital care managers, Minnesota Department of Health, Minnesota Nurses Association, county social workers, and insurance payers. One of the primary goals was to develop and attempt to validate a QT that reliably allocated home care nursing hours based on medical complexity and skilled nursing needs. The first QT to assign nursing hours was developed in 2012. Due to a home care nursing shortage, the QT was modified in 2016 and resulted in a decrease in assigned nursing hours.

The proposed guidelines were shared with nursing agencies, hospitals, discharge planners, and other stakeholders in Minnesota involved in the care of CMC. The QT attempts to objectively standardize recommendations for home care nursing hours based on a medical complexity and skilled nursing interventions. Collaboration among home care nursing agencies resulted in assignment of nursing hours for various nursing tasks. During the development and testing of the tool, feedback from nursing agencies resulted in increases or decreases in recommended nursing hours. This was based on real-life experiences, the number, type, and frequency of nursing interventions.

## Hourly Nursing Qualification Tool

There are three tables that comprise the QT: Primary Indication for Nursing (Table 1) was developed to incorporate the organ systems of patients commonly referred for home care nursing hours. Adjunctive Nursing Hours (Table 2) and Skilled Nursing Treatments (Table 3) were developed to recognize the workload associated with skilled nursing interventions and treatments. A rationale for additional nursing hours may also be justified for posthospital care with a new complex diagnosis (Auger et al., 2019). Compiling the results from the tables provides recommended home care nursing hours.

**Table 1.** Primary Indication for Nursing

Organ System	Description	Clinical	Nursing Hours/Week
			Qualification Tool
Pulmonary	Mechanical ventilation, invasive and noninvasive (excludes obstructive sleep apnea)	Vent dependent 24 hr/day	84
		12-23 hr/day	63
		1-11 hr/day	42
	Tracheostomy-dependent, no mechanical ventilation	Unstable airway, decannulation is life threatening	63
		Stable airway, decannulation not immediately life threatening	35
	Airway management without mechanical ventilation or tracheostomy	Inability to maintain patent airway independently, frequent suctioning and repositioning, high risk for sentinel event	63
		Frequent respiratory infections and symptoms, aspiration risk	30
		Frequent respiratory assessment and monitoring for potential infection and aspiration	0
Cardiac	Unstable cardiac condition	Life-threatening episodes past 45 days or daily intervention to prevent life-threatening episodes (such as PRN medications or other interventions for acute arrhythmia or arrest)	14
Central nervous system (CNS)	Unstable neurological disorder	Seizures ≥3/week in past 45-60 days that require interventions, or grand mal drop or intractable seizure that affects daily function	21
		Seizures ≥1-2 days in past 45-60 days that require interventions and affect daily function, or neuromuscular disorder requiring skilled nursing interventions (autonomic dysreflexia, spina bifida, muscular dystrophy, etc.)	10.5
Gastrointestinal	Complex gastrointestinal disorder	Combination of parenteral and enteral nutritional requirement with ongoing assessment of fluid losses requiring adjustments	42
End of life	End-of-life care	Patient requiring care related to end of life	42

**Table 2.** Adjunctive Nursing Hours

Clinical	Risk Level	Nursing Hours/Week
		Qualification Tool
**Skilled Nursing Treatments** (see Table 3 to calculate risk level)	
Registered nurse (RN) or licensed practical nurse (LPN) required to complete (Table 3) Select only one, if indicated	High	21
	Medium	9
	Low	5
**Medications**	
Continuous intravenous or oxygen therapy for unstable condition (rehospitalization within 1 hr, if interrupted)	High	21
Skilled intervention to perform/change medication regimen with physician order	Medium	14
Low risk for drug interaction, routine scheduled or PRN medication	Low	0
**Hospitalizations**	
Posthospital care with new complex diagnosis; transient need for increased hours	High	10.5
Posthospital care following unplanned hospitalization needing transient increased care for a preexisting condition	Low	3.5
**Organ Systems/Interventions**	
Performed by RN/LPN for comorbidities Organ systems involved ≥3	High	5
Organ systems involved ≥2	Low	0
**Clinical**	
Tracheostomy, nasopharyngeal, or oral suctioning by RN/LPN that requires nursing observation/intervention to maintain patient airway	
Hourly or greater	High	10
q 2-4 hr	Medium	5
q 5-8 hr	Low	1.5
**Feeding**	
Parenteral and enteral, adjustments based on weaning or fluid imbalance/Intake & Output	High	24.5
Nasogastric feeds or complex feeding regimen. History of aspiration/swallowing disorder	Medium	5
Gastrojejunal drip feeds	Low	5
**Caregiver**	
Complex social, family, and environmental situations; physical limitations of caregiver; single parent with other children <5 years; minimal trained backup caregivers	High	2
Social, family, environmental situations are not complex	Low	0
**Mobility**	
Total dependence; requires 1-2 people and/or assistive equipment for safe transfer and positioning	High	5
Assistance required but patient able to bear weight with/without assistive devices	Low	2
**Cognition**	
Altered cognition creates safety issue for patient; requires constant observation or supervision due to memory, decision making, judgment	High	3.5
No issues with altered cognition requiring some intermittent observation or supervision due to problems with memory, decision making, or judgment	Low	0
**Behavior **	
Behavior that creates a safety issue for nurse or patient. Interventions and time required to maintain a safe environment, prevent impediment of care, educate, and minimize noncompliance and violent outbursts	High	3.5
Intermittent behavioral issues warranting interventions	Low	0

**Table 3.** Skilled Nursing Treatments (Nursing Hours Added to Table 2)

Risk Level	Nursing Hours/Week	Interventions
**High**	21	Dialysis (performed by nurse) Second- or third-degree burn care (>10% of the body) 3 or more medium complex treatments listed under Medium (below) Vascular catheter (accessed by nurse)
**Medium**	9	Tracheostomy care/change Chemotherapy (administered by nurse) Intermittent urinary catheterization (insertion/maintenance by nurse) Open stage 3 or 4 decubitus ulcer or open surgical site wound care Second- or third-degree burn care (<10% of the body) Wound vac (managed by nurse)
**Low**	5	Scheduled pulmonary toileting program (includes respiratory vest, chest physiotherapy, insufflation-exsufflation device) Turning/repositioning program Ileostomy, colostomy care External catheter (condom catheter; insertion/maintenance by nurse) Indwelling catheter care, bladder irrigation Bowel program, enemas Stasis ulcer Intake/output measurement

### Table 1: Primary Indication for Nursing

Baseline nursing hours are determined by selecting one of the primary organ systems listed in Table 1 that requires the most skilled nursing interventions. In the initial 2012 tool, three organ systems were adopted (pulmonary, cardiac, and central nervous system). Gastrointestinal was added when the tool was modified in 2016, prompted by increasing referrals for patients with intestinal failure needing complex feeding regimens, central venous catheters for parenteral nutrition, and the need to closely monitor fluid status. An additional provision was also made in 2016 for end-of-life care. Families of children who received long-term care nursing requested to keep their HCNs rather than using hospice nurses when their child's situation warranted hospice or palliative care (Lindley et al., 2016).

The pulmonary section in Table 1 is divided into three subsets based on invasive or noninvasive mechanical ventilation, presence of a tracheostomy without invasive ventilation, or the need for close supervision to maintain a patent airway in the absence of an artificial airway. For example, noninvasive ventilation is increasingly used to manage infants and young children requiring chronic respiratory assistance (McDougall et al., 2013). Vigilance, as well as skilled nursing interventions, are necessary for a safe care plan. These young patients are unable to readjust or clear vomitus from their face mask and incapable of alerting caregivers during acute respiratory decompensation.

Another narrative addressed in the pulmonary section is a child with a tracheostomy. These children are managed with an artificial airway. Unrecognized plugging or accidental decannulation of the tracheostomy tube that may have rapidly fatal consequences and preventable deaths in a population of children with tracheostomies has been recently reviewed at 11% (Foy et al., 2020). Another description in the pulmonary section relates to children with upper airway obstruction that can be managed without invasive or noninvasive mechanical ventilation. Severe cases of Pierre-Robin sequence and Beckwith-Wiedemann syndrome are examples where nursing interventions may include repositioning and naso-oropharyngeal suctioning to decrease the risk for airway obstruction.

### Table 2: Adjunctive Nursing Hours

This table identifies additional hours of nursing care to be added to the baseline hours previously established in Table 1, or to assign nursing hours to patients who fall outside the primary indications in Table 1. Table 2 describes skilled treatments/interventions that would generally require an registered nurse (RN) or licensed practical nurse (LPN) to perform and stratifies them at a “risk level” of high, medium, or low based on Table 3. Only one row from each of the descriptors in Table 2 is selected to calculate recommended home care nursing hours. If the descriptors listed in Table 2 do not meet the patient's profile, then no hours are assigned from that row. For example, a cognitively intact wheelchair-bound 16-year-old would not receive hours under cognition, but would receive hours under mobility. The Caregiver section in Table 2 addresses complex social, family, environmental situations, physical limitations of the caregiver, as well as the presence of other children <5 years of age in the home. For example, this section may recommend additional hours for a single parent with multiple children in the household.

### Table 3: Skilled Nursing Treatments

This table recommends additional nursing hours based on specific nursing interventions, procedures, or treatments stratified as high, medium, or low risk. These skilled nursing hours 21, 9, or 5 hours, respectively, are added to the top of Table 2 under Skilled Nursing Treatments. A patient not requiring interventions listed in Table 3 would have no additional nursing hours added to the Skilled Nursing Treatments section at the top of Table 2.

### Using the QT

The initial approach to using the QT is to assign a primary targeted diagnosis from one of the rows in Table 1. After further review of medical records, additional nursing hours are identified and assigned in Table 2. If the patient has specific skilled nursing treatments identified in Table 3, those hours are added into the first row of Table 2. The summation of hours from Tables 1 and 2 provides the recommended number of nursing hours/week. On occasion, there are patients who do not have a primary indication for nursing (Table 1). In those instances, only Tables 2 and 3 are used to calculate nursing hours. Completing the QT involves patient assessment and review of the medical record to assign and calculate nursing hours, which takes approximately 1 hour to complete.

After identifying there are adequate HCNs to accept and staff a new patient referral, the nursing agency completes the QT and shares results with the patient's physician. A physician's order with medical documentation for proposed nursing hours is forwarded to the insurance company for authorization. Allocated home care nursing hours are reviewed every 60 days or as needed (concurrently with the 485 plan of care) to recommend changes in assigned hours if there is a change in medical status.

## Case Studies

The following case studies illustrate how the Minnesota QT is used to calculate and recommend nursing hours. Each case begins with a brief history followed by a list of the child's direct nursing needs. Case studies were adapted and modified from real-life cases. Modifications were made to illustrate the range of complexities and feasibility of the QT. The authors (LP, CL) collaborated on the case studies using the QT and the recommended nursing hours for each case was a consensus.

### Case Study 1

A 9-month-old former 28-week female ready for hospital discharge is referred for home care. This child has a tracheostomy, ventilator, and is oxygen-dependent 24-hours-per-day for chronic respiratory failure secondary to bronchopulmonary dysplasia and tracheomalacia. Additional gastrointestinal comorbidities include retching, gagging, and history of aspiration treated with continuous drip feedings via gastrostomy. Medications include nebulized albuterol and budesonide twice daily (BID), which are escalated when ill, chlorothiazide and lansoprazole BID, vitamins daily, and three other “as needed” medications. See Table 4 for determining nursing hours.

**Table:** Organ Systems/Interventions

Respiratory	Adjust oxygen to keep saturations >90% Generally requires sterile suctioning at least every hour and PRN Tracheostomy tube change every 2 weeks and PRN Chest physiotherapy PRN
Gastrointestinal	Continuous GT feedings Continuous venting of GT to Farrell valve bag
Integumentary	Trach and GT stoma cares BID and PRN
Musculoskeletal	Physical, speech, and occupational prescribed therapies performed by nurse

**Table 4.** Determining Nursing Hours Using Qualification Tool for Case Study 1

Table	Organ System	Clinical	Risk Level	Nursing Hours/Week
1	Pulmonary	24-hr-per-day ventilator-dependent child		84
**Total (Table 1)**				**84**
2		Skilled Nursing Treatments (Table 3)	Medium	9
		Medications	Medium	14
		Hospitalizations	High	10.5
		Organ systems/interventions (≥3)	High	5
		Clinical (trach suctioning q 1 hr)	High	10
		Feeding (complex, aspiration risk)	Medium	5
		Mobility (1-2 people for safe transfer/positioning)	High	5
**Total (Table 2)**				**58.5**
**Total recommended (sum of Tables 1 & 2)**				**142.5**
**Recommendation:** Initially discharged with 24 hr/day nursing care for 2 weeks posthospital discharge then decreased to 20 hr/day. Hours are reevaluated every 60 days or significant change in medical status.				

### Case Study 2

A 9-year-old, 21.8 kg male with De Barsy syndrome, osteoporosis, chronic obstructive pulmonary disease, gastroesophageal reflux disease with history of aspiration pneumonia, frequent respiratory symptoms and infection, gastrojejunostomy feeding tube, and frequent coughing and gagging episodes requires nasal and oropharyngeal suctioning to maintain a patent airway. Medications include nebulized albuterol and budesonide BID, calcium carbonate, lansoprazole, ergocalciferol, polyethylene glycol 3,350 daily, gabapentin three times daily, and nebulizer treatments are increased to 6 ×/day as needed with acute respiratory symptoms. There have been no recent hospitalizations while the patient receives extended hours of nursing care. See Table 5 for determining nursing hours.

**Table:** Organ Systems/Interventions

Respiratory	Adjust oxygen to keep sats >90% Nasal and oral suctioning TID and PRN Bronchial drainage chest percussion TID and PRN
Gastrointestinal	Continuous jejunal feedings Gastrostomy with continuous venting to Farrell valve bag
Integumentary	Tracheostomy stoma cares BID and PRN Gastrostomy stoma cares daily and PRN
Musculoskeletal	Therapies directed by physical, speech, and occupational therapists

**Table 5.** Determining Nursing Hours Using Qualification Tool for Case Study 2

Table	Organ System	Clinical	Risk Level	Nursing Hours/Week
1	Pulmonary	Frequent respiratory infections and symptoms, aspiration risk		30
**Total (Table 1)**				**30**
2		Skilled Nursing Treatments (Table 3)	Low	5
		Medications	Medium	14
		Organ systems/interventions (≥3)	High	5
		Clinical (suctioning three times a day and as needed)	Low	1.5
		Feeding (complex, aspiration risk)	Medium	5
		Mobility (1-2 people for safe transfer/positioning)	High	5
		Cognition	Low	0
**Total (Table 2)**				**35.5**
**Total recommended (sum of Tables 1 & 2)**				**65.5**
**Recommendation:** Nursing hours 65.5 hr/week. Hours are reevaluated every 60 days or significant change in medical status.				

### Case Study 3

A 2-year-old, 12.3 kg female has been hospitalized since birth due to complications from 28-week premature birth and necrotizing enterocolitis. Despite several serial transverse enteroplasty procedures, she remains on parenteral nutrition through a broviac catheter as well as daytime bolus and nocturnal continuous drip feedings through a gastrostomy tube. She also has variable inspiratory stridor and is discharging home with parents. Discharge diagnoses include short bowel syndrome, malabsorption, total parenteral nutrition dependent, feeding difficulties, and congenital laryngomalacia. Medications include albuterol HFA and beclomethasone inhalers 2 puffs QID. As needed medications include diphenhydramine, ondansetron, simethicone, acetaminophen, and topical nystatin and hydrocortisone. There are no caregiver, cognition, or behavior concerns. See Table 6 for determining nursing hours.

**Table:** Organ Systems/Interventions

Gastrointestinal	Continuous GT feedings at night with bolus feedings during the day, PRN venting of Farrell valve bag by syringe or gravity, input and output monitoring
Vascular	Total parenteral nutrition administration, central line assessment and care, dressing changes
Respiratory	Assessment of respiratory status and medication administration
Integumentary	GT stoma care
Musculoskeletal	Therapies directed by physical, speech, and occupational therapists

**Table 6.** Determining Nursing Hours Using Qualification Tool for Case Study 3

Table	Organ System	Clinical	Risk Level	Nursing Hours/Week
1	Gastrointestinal	Postsurgical malabsorption requiring hyperalimentation		42
**Total (Table 1)**				**42**
2		Skilled Nursing Treatments (Table 3)	High	21
		Medications	Medium	14
		Hospitalizations (posthospital with new diagnosis)	High	10.5
		Organ systems/interventions (≥3)	High	5
		Feeding (parenteral and enteral, requirement for adjustments based on weaning, fluid imbalance, input/output)	High	24.5
		Caregiver	Low	0
		Cognition	Low	0
		Behavior	Low	0
**Total (Table 2)**				**75**
**Total recommended (sum of Tables 1 & 2)** Nursing hours 117 hr/week. Hours are reevaluated every 60 days or significant change in medical status.				**117**
**Recommendation:** Nursing hours 117 hr/week. Hours are reevaluated every 60 days or significant change in medical status.				

### Case Study 4

A 10-month-old, 2.47 kg female is referred for home care nursing at hospital discharge. An EEG confirmed up to 200 subclinical seizures per day of unknown etiology. Patient has visible seizure activity that requires administration of rescue seizure medication up to five times per week. Unstable thermoregulation is present and an elevated body temperature exacerbates her clinical seizures. Seizures may occur with bottle feeding, aspiration, vomiting, desaturation, and a seizure protocol is initiated for seizures lasting longer than 5 minutes. Bottle feedings are supplemented by gastrostomy feedings. Medications administered via GT include lacosamide 12.2 mg BID, levetiracetam 85 mg BID, pediatric multivitamin qd, and topiramate 19 mg BID. Seizure protocol includes diazepam 2.5 mg rectally PRN. See Table 7 for determining nursing hours.

**Table:** Organ Systems/Interventions

CNS/neuroepilepsy, unspecified, intractable, other reduction deformities of the brain	Seizure protocol: emergency administration of diazepam rectally every 10 min PRN for seizures lasting >5 min Temperature assessment q 4 hr: administer antipyretics q 4-6 as needed for temp >101.5°F
Gastrointestinal	Feeding difficulties Gastrostomy tube
Respiratory	Aspiration precautions
Integumentary	GT stoma care daily and PRN
Musculoskeletal	Therapies provided under direction of physical, speech, and occupational therapists

**Table 7.** Determining Nursing Hours Using Qualification Tool for Case Study 4

Table	Organ System	Clinical	Risk Level	Nursing Hours/Week
1	CNS	Seizures ≥3/week in past 45 to 60 days that require interventions, or grand mal drop or intractable seizure that affects daily function		21
**Total (Table 1)**				**21**
2		Skilled Nursing Treatments (Table 3)	n/a	0
		Medications: intervention to administer/change regimen with physician order	Medium	14
		Hospitalizations: posthospital care new complex diagnosis	High	10.5
		Organ systems/interventions (≥3)	High	5
		Feeding: Nasogastric or complex feeding regimen, history of aspiration, swallowing dysfunction	Medium	5
		Mobility (1-2 people for safe transfer/positioning)	High	5
**Total (Table 2)**				**39.5**
**Total recommended (sum of Tables 1 & 2)**				**60.5**
**Recommendation:** Nursing hours 60.5 hr/week. Hours are reevaluated every 60 days or significant change in medical status.				

## Validation of the QT

To validate the QT, anonymous surveys via SurveyMonkey^®^ were sent to three different groups. The first survey was dispatched only to nursing staff who use the QT to recommend nursing hours. This included case managers at Pediatric Home Service (Roseville, Minnesota), nurse managers at other home care agencies, discharge coordinators/case managers at hospitals, and one pediatric pulmonary clinic-based home care manager. The survey included the four case studies, the current 2016 QT, and a request to calculate nursing hours. A second query asked “ease of use” of the QT based on a 5-point Likert scale. This study was deemed exempt by Children's Minnesota Institutional Review Board.

A second anonymous survey was sent to the parents of children who received home care nursing hours allocated by the 2016 QT and included two queries. Parents were asked to select the range of nursing hours/week they were receiving ≥2 weeks postinitial hospital discharge. As many families go home with more nursing hours during the first 1 to 2 weeks posthospital discharge, this identified baseline nursing hours after the transition period. The second query, based on this same time period, asked parents to evaluate their perception of the nursing hours they were receiving based on a 5-point Likert scale. The third anonymous survey using a 5-point Likert scale was applied to HCNs working in homes where nursing hours were determined by the 2016 QT.

## Results

**Figure 1.**
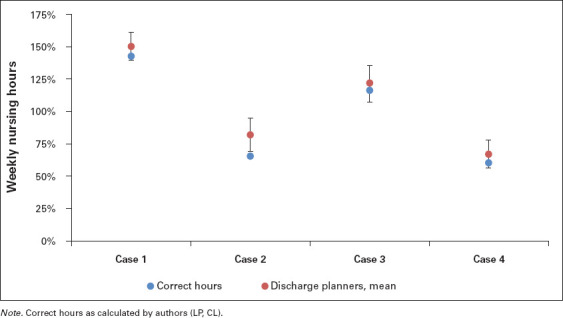
Comparison of Calculated Nursing Hours

In the first group, 8 out of 18 healthcare workers responded to the survey. The mean and range for nursing hours calculated by discharge planners, as compared with calculated hours from two of the authors (LP, CL), is shown in Figure 1. The “percentage difference” in calculated hours in Case Studies 1 through 4 were 5.7, 24.9, 4.1 and 11.2, respectively, including mean and median hours (Table 8). Discharge planners rated “ease of use” of the QT at a mean of 3.6 based on a 5-point Likert scale with 5 very easy to use and 1 very difficult to use.

**Table 8.** Calculation of Mean, Median, and Percentage Difference of Nursing Hours

	Case 1	Case 2	Case 3	Case 4
Correct hours	142.5	65.5	117.0	60.5
Discharge planners, mean	150.7	81.8	121.8	67.3
Discharge planners, median	144.5	82.3	117.0	61.8
Standard deviation	15.4	18.3	20.4	15.8
Number of respondents	8	8	8	8
Standard error	5.5	6.5	7.2	5.6
Error bar	10.7	12.7	14.1	10.9
Difference	8.2	16.3	4.8	6.8
% difference	5.7%	24.9%	4.1%	11.2%

*Note*. Correct hours as calculated by authors (LP, CL).

**Figure 2.**
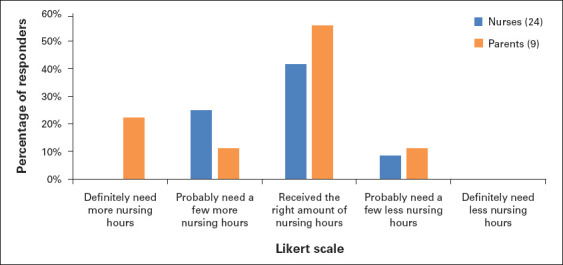
Parent/Nurse Perception of Nursing Hours

The parent survey was answered by 9 out of 17 parents. Assigned nursing hours 2 weeks or more beyond hospital discharge identified that 4 out of 9 patients were receiving 41-80 hours/week and 5 out of 9 were receiving 121-168 hours/week. Parents' perceptions of allocated nursing hours are shown in Figure 2 alongside responses from 24 of 28 surveyed HCNs. There was no significant difference in Likert scores between parents and nurses regarding allocated nursing hours. The hours calculated by the QT met the perceived need for weekly nursing hours of parents and nurses 56% and 42% of the time, respectively. The need for additional nursing hours was expressed by 33% and 50% of parents and nurses, respectively. In general, HCNs' perception of nursing hours paralleled that of parents with a trend for nurses to perceive a need for additional home care nursing hours.

## Discussion

Validation of the QT included an objective calculation of recommended nursing hours/week based on four hypothetical case studies. We were unable to identify other QTs for assigning nursing care hours in the literature. There was a very close correlation between median nursing hours calculated by discharge planners and the authors (LP, CL) except for Case 2 which had a 25% difference (Table 8). Case 2 provides insight into the need for further refinement of the QT. The interpretation of Case 2 by participating discharge planners may have included a subjective analysis regarding “Clinical” airway patency and “Cognition.” We speculate additional hours were recommended by the discharge planners based on those descriptors and accounted for the greater percentage difference. Having direct access to the medical documentation of a patient rather than a presented case study could minimize the subjectivity and variations as found with Case 2.

Although the QT met the parents' and nurses' perceived need for nursing hours 56% and 42% of the time, there was still a significant perceived need for more nursing hours by both groups. A published report identified that physicians and nurses believed families with children on home mechanical ventilation should receive more nursing hours, at least during the transition to home (Sobotka et al., 2020). A Midwest study on children receiving palliative home care nursing found a 40-hour-per-week difference between allotted versus received hours and attributed this to a home care nursing shortage (Weaver et al., 2018). Also, a workforce scarcity in Minnesota contributed to the modification of the initial 2012 QT to the current 2016 QT with a reduction in assigned nursing hours.

Technology-dependent children are a high-risk population and offer a unique challenge to successfully transition from hospital to home. As this population increases, one hurdle to overcome is a shortage of HCNs (Maynard et al., 2019) and a lack of stability for nursing services to meet the needs of the patient and family (Nageswaran & Golden, 2017b). Another barrier for home health services for children is the lack of training for nurses (Nageswaran & Golden, 2017a). HCNs are an important community healthcare resource and priorities for staffing should be on facilitating a safe hospital discharge and the initial stabilization at home for CMC. After the initial transition to home, there tends to be a reduction in the number of assigned home care nursing hours. Standardizing the allotment of nursing hours optimizes availability of this scarce resource. Variations across state lines impact access to pediatric home care nursing, home care nursing hours, and contributes to healthcare disparities (Rasooly et al., 2020). Proposals suggest there should be federal standards for Medicaid home-based services (Ollove, 2021). Nursing agencies need to be held accountable and reduce home care nursing hours as a patient's clinical status and acuity improve, and need for skilled nursing care decreases. In contrast, patients with a worsening clinical status may warrant a transient increase in nursing hours.

In contrast, guidelines have been proposed for adult home healthcare (U.S. Department of Health and Human Services, Centers for Medicare & Medicaid Services, 2020), but gaps exist in home healthcare for CMC (Foster et al., 2019; Simpser & Hudak, 2017). Families with children that require home care nursing after discharge from the hospital for the first time need to understand and assume responsibility for medical care in the home, knowing that over time home care nursing hours may be reduced. The role of an HCN immediately after discharge goes well beyond direct care of the child. An attentive HCN instills confidence and competence in the family for the care of their child. HCNs facilitate independence by educating and empowering families in assessment, skilled interventions, and other complex health-related tasks needed to care for their child. Ultimately, this allows some skilled nursing hours to be reassigned to families during their transitional period from hospital to home without compromising properly trained families with established nursing home care.

### Study Limitations

Access to home care nursing varies from state-to-state and is affected by health insurance and home care nursing shortages. This study was limited to one geographical region, Minnesota, where discharge planners and nursing agencies were familiar with the QT. The goal was to develop an objective method to allocate nursing hours recognizing that interpretation of a patient's medical record still leaves room for subjectivity. Some of the percentage differences in case study hours may reflect that the discharge planners who participated only had access to hypothetical case studies and not actual medical records. As the 2016 QT has only been available for 4 years in Minnesota, a smaller number of families were available to survey, and just over 50% of surveyed parents responded. As the surveys were anonymous, we cannot compare responses from parents and nurses within the same household. A smaller percentage of end-users of the QT responded to our survey, which could create bias. Validation of the QT can be measured objectively by comparing interuser variability in calculating nursing hours, and although very close in three out of four case studies, there is a potential need for further refinement of the QT. Limitations of standardized QTs to meet the needs of patients and families (Agrawal, 2015) have been reviewed.

## Conclusion

A QT was developed in response for a need to equitably triage home care nursing hours in the face of a pediatric HCN shortage. Minnesota home care nursing agencies and discharge planners use the QT to objectively and consistently recommend home care nursing hours based on medical complexity and need for skilled nursing interventions. Over 50% of surveyed parents felt the assigned nursing hours, based on this QT, matched their needs. This sentiment was paralleled by the majority of surveyed HCNs. Further refinement and acceptance of this QT could standardize eligibility requirements for pediatric nursing hours across state lines. Emphasis should also be placed on empowering families at the time of hospital discharge to cope with the realities of a shrinking home healthcare workforce.
